# Complete genome sequence of the type strain *Ampullimonas aquatilis* LMG 28208^T^

**DOI:** 10.1128/mra.00384-25

**Published:** 2025-07-07

**Authors:** William M. Moe, Luciana Albuquerque, Debajyoti Deb

**Affiliations:** 1 Department of Civil and Environmental Engineering, Louisiana State University 5779, Baton Rouge, Louisiana, USA; 2 CNC-UC – Center for Neuroscience and Cell Biology, University of Coimbra, UC-Biotech, BiocantPark 530237, Cantanhede, Coimbra District, Portugal; 3 CIBB – Center for Innovative Biomedicine and Biotechnology, University of Coimbra, UC-Biotech, BiocantPark 649195 , Cantanhede, Coimbra District, Portugal; University of Southern California, Los Angeles, California, USA

**Keywords:** *Betaproteobacteria*, *Alcaligenaceae*, *Burkholderiales*, nitrogen fixation, denitrification

## Abstract

*Ampullimonas aquatilis* LMG 28208^T^ is a Gram-stain-negative, nitrogen-fixing bacterium of the order *Burkholderiales*. Here, we report complete sequences for the type strain 3,600,201 bp chromosome (49.84 mol% G + C) and single, circular 75,931 bp plasmid. These will facilitate phylogenetic comparisons and aid future efforts to assess the role of *Ampullimonas* in the environment.

## ANNOUNCEMENT

The nitrogen-fixing, nitrate-reducing, type strain *Ampullimonas aquatilis* B15.09-116^T^ (=LMG 28208^T^) was isolated from bottled mineral water in Portugal (1). Until now, no *Ampullimonas* genome sequences were available for taxonomic or functional analysis.

*Ampullimonas aquatilis* LMG 28208^T^ was obtained from the BCCM/LMG culture collection (Ghent, Belgium). Cells grown in R2A broth (25°C, 12 days) were harvested by centrifugation (3,000 × *g*, 30 min). DNA was isolated using a GenElute Bacterial Genomic DNA Kit (Sigma-Aldrich). The same DNA prep was used for both Oxford Nanopore Technologies (ONT) and Illumina libraries. For ONT, a library prepared using a ligation sequencing kit (SQK-LSK114) was sequenced at EzBiome (Gaithersburg, MD) on an ONT MinION Mk1C device with R10.4.1 flow cell (2). No shearing or size selection was performed prior to library preparation. Basecalling (SUP model) and adapter/barcode removal were performed using Dorado basecall server v7.4.12 (ONT). Low-quality reads (bottom 5%) from the initial 63,710 reads were filtered using Filtlong v0.2.1 (github.com/rrwick/Filtlong). Filtered reads (*N*_50_ = 8,040 bases) were assembled using Flye v2.9.5 (3) with –nano-hq assembly mode.

Illumina sequencing library construction employed a NEBNext Ultra Library Prep Kit for Illumina, and paired-end sequencing (2 × 150  bp) was performed using a NextSeq 2000 system (Illumina). BBDuk in BBMap v39.13 (sourceforge.net/projects/bbmap/, settings as described [4]) was used to trim and filter low-quality sequences from the initial library of 6,748,948 PE reads. Filtered Illumina reads were employed to polish the ONT assembly using Polypolish v0.6.0 (5). The final assembly mean coverage depth was 71× ONT and 562× Illumina. Annotation was performed using NCBI Prokaryotic Genome Annotation Pipeline v6.9 (6). All software employed default parameters except where noted otherwise.

The *Ampullimonas aquatilis* LMG 28208^T^ genome comprises a circular 3,600,201 bp chromosome (49.84 mol% G + C) and a single, circular 75,931 bp plasmid (49.59 mol% G + C). CheckM v1.4.0 (7) analysis (*Betaproteobacteria* marker set) estimated 99.37% completeness and 0.0% contamination. In total, the genome contains 3,437 predicted genes, including several annotated as encoding proteins associated with nitrogen fixation and nitrate reduction and two identical copies each of 5S, 16S, and 23S rRNA genes.

Pairwise comparisons revealed that both genome 16S rRNA gene copies (locus_tags: ACMYR3_00005 and ACMYR3_12900) and *nifH* gene (ACMYR3_04175) differ from sequences reported previously (JX458401.1, KT964778.1), with one mismatch and nine indels in the 16S rRNA and eight mismatches in the *nifH* gene. To resolve these discrepancies, nearly complete 16S rRNA genes and partial *nifH* genes from strain LMG 28208^T^ and strain B15.09-116^T^ (obtained from the CNC-UC private collection where it was first isolated) were PCR-amplified using universal bacterial primers 27f/1,525r (8) and PolF/PolR (9), respectively. Reactions employed a *Taq* PCR Kit (NEB E5000S) and ABI Veriti thermocycler with thermal protocols described previously (8, 9). Amplicons purified using ExoSAP-IT PCR Product Cleanup were bidirectionally sequenced (8, 9) using BigDye Terminator v3.1 chemistry on an ABI 3730XL DNA Analyzer and manually assembled using BioEdit v7.0.5.3 (10). The resulting nearly-complete 16S rRNA gene and partial *nifH* gene sequences were identical across both strains and matched the genome. Genome 16S rRNA and *nifH* gene sequences should replace previous sequences (JX458401.1, KT964778.1) which contain errors.

## Data Availability

The complete genome sequence of Ampullimonas aquatilis LMG 28208^T^ is deposited in GenBank under accession numbers CP183834 and CP183835 (BioProject accession number PRJNA1220035). Oxford Nanopore, Illumina, and Sanger sequencing reads for strain LMG 28208^T^ are available from the NCBI Sequence Read Archive (SRA) under accession numbers SRR32258265, SRR32258264, and SRR32956526–SRR32956527, respectively.
